# Intestinal parasitic infections in schoolchildren in different settings of Côte d’Ivoire: effect of diagnostic approach and implications for control

**DOI:** 10.1186/1756-3305-5-135

**Published:** 2012-07-06

**Authors:** Jean T Coulibaly, Thomas Fürst, Kigbafori D Silué, Stefanie Knopp, Dimitri Hauri, Mamadou Ouattara, Jürg Utzinger, Eliézer K N’Goran

**Affiliations:** 1 Department of Epidemiology and Public Health, Swiss Tropical and Public Health Institute, P.O. Box, CH-4002, Basel, Switzerland; 2 University of Basel, P.O. Box, CH-4003, Basel, Switzerland; 3 Unité de Formation et de Recherche Biosciences, Université de Cocody, 22 BP 770, Abidjan 22, Côte d’Ivoire; 4 Centre Suisse de Recherches Scientifiques en Côte d’Ivoire, 01 BP 1303, Abidjan 01, Côte d’Ivoire

## Abstract

**Background:**

Social-ecological systems govern parasitic infections in humans. Within the frame of assessing the accuracy of a rapid diagnostic test for *Schistosoma mansoni* in Côte d’Ivoire, three different endemicity settings had to be identified and schoolchildren’s intestinal parasitic infection profiles were characterized.

**Methods:**

In September 2010, a rapid screening was conducted in 11 schools in the Azaguié district, south Côte d’Ivoire. In each school, 25 children were examined for *S. mansoni* and *S*. *haematobium.* Based on predefined schistosome endemicity levels, three settings were selected, where schoolchildren aged 8–12 years were asked to provide three stool and three urine samples for an in-depth appraisal of parasitic infections. Triplicate Kato-Katz thick smears were prepared from each stool sample for *S. mansoni* and soil-transmitted helminth diagnosis, whereas urine samples were subjected to a filtration method for *S. haematobium* diagnosis. Additionally, a formol-ether concentration method was used on one stool sample for the diagnosis of helminths and intestinal protozoa. Multivariable logistic regression models were employed to analyse associations between schoolchildren’s parasitic infections, age, sex and study setting.

**Results:**

The prevalences of *S. mansoni* and *S. haematobium* infections in the initial screening ranged from nil to 88% and from nil to 56%, respectively. The rapid screening in the three selected areas revealed prevalences of *S. mansoni* of 16%, 33% and 78%. Based on a more rigorous diagnostic approach, the respective prevalences increased to 33%, 53% and 92% *S. haematobium* prevalences were 0.8%, 4% and 65% (rapid screening results: 0.0%, 0.0% and 54%). Prevalence and intensity of *Schistosoma* spp., soil-transmitted helminths and intestinal protozoan infections showed setting-specific patterns. Infections with two or more species concurrently were most common in the rural setting (84%), followed by the peri-urban (28%) and urban setting (18%).

**Conclusions:**

More sensitive diagnostic tools or rigorous sampling approaches are needed to select endemicity settings with high fidelity. The observed small-scale heterogeneity of helminths and intestinal protozoan infections has important implications for control.

## Background

Intestinal parasitic infections (e.g. helminths and pathogenic intestinal protozoa) are of considerable public health importance, particularly in developing countries. For example, the global burden caused by soil-transmitted helminthiasis (infections with *Ascaris lumbricoides*, hookworm and *Trichuris trichiura*) is estimated at 39 million disability-adjusted life years (DALYs), whereas the burden due to schistosomiasis (mainly *Schistosoma mansoniS. haematobium* and *S. japonicum*) is estimated at 4.5 million DALYs [1,2]. Amoebiasis due to infections with the intestinal protozoon *Entamoeba histolytica* results in 40,000–100,000 deaths each year [3], and giardiasis due to *Giardia intestinalis* might affect 200 million people per annum [4]. However, the burden of pathogenic intestinal protozoan infections in terms of DALYs remains to be determined, which is a challenge due to the paucity of up-to-date epidemiological data [5].

Parasitic infections are governed by behavioural, biological, environmental, socioeconomic and health systems factors. Local conditions, including access to and quality of domestic and village infrastructure, economic factors such as disposable income, employment and occupation, and social factors such as education, influence the risk of infection, disease transmission and associated morbidity and mortality [6-8].

As a preparatory step for a rigorous assessment of the accuracy of a new rapid diagnostic test for *S. mansoni* in Côte d’Ivoire, we aimed to identify a low prevalence (10–24%) and a moderate prevalence (25–49%) setting for *S. mansoni*, and a third setting where *S. mansoni* and *S. haematobium* co-exist. While in the initial screening only single stool and urine samples were collected from each individual. In the main study, the diagnosis was based on multiple stool and urine examinations to more accurately characterize schoolchildren's intestinal parasitic infection profiles in the selected study areas. Implications of our findings for the design, implementation and monitoring of intestinal parasite control strategies in Côte d’Ivoire are discussed.

## Methods

### Ethical considerations

This study was approved by the institutional research commission of the Swiss Tropical and Public Health Institute (Basel, Switzerland) and received clearance from the ethics committees in Basel (EKBB, reference no. 377/09) and Côte d’Ivoire (reference no. 1993 MSHP/CNER). The local authorities (i.e. village chiefs, school directors, teachers and medical staff) were informed about the objectives and procedures of the study. Literate parents and legal guardians of eligible schoolchildren were given an information sheet, whereas those who were illiterate were informed in lay terms by the teachers. Written informed consent was obtained from parents/guardians. Children assented orally. Participation was voluntary, and hence, children could withdraw at any time without further obligations.

At the end of the study, all participating schools were offered free treatment regardless of the infection status of the children. Praziquantel, manufactured by Bayer (single 40 mg/kg oral dose using a dose-pole) and albendazole, obtained from GlaxoSmithKline (single 400 mg oral dose), were administered by medical staff.

### Selection of study settings

**Figure 1:**
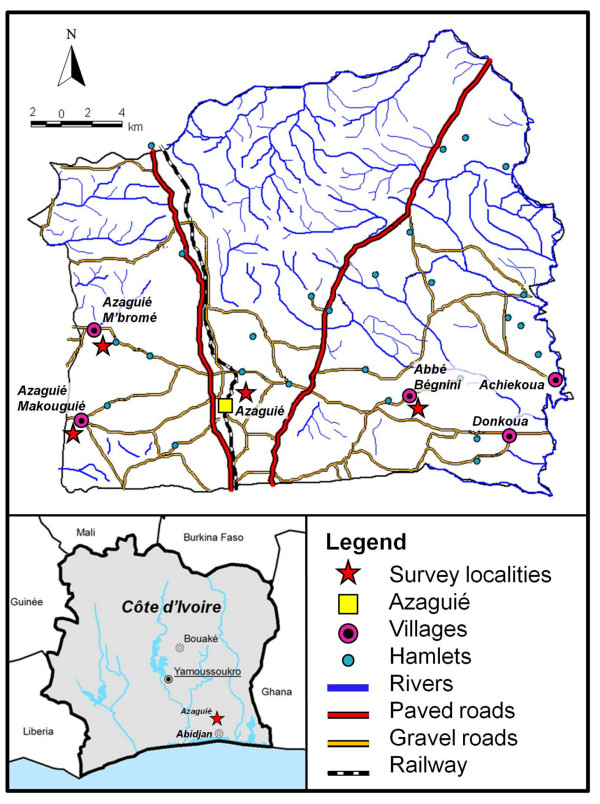
**Map showing the district of Azaguié in south Côte d’Ivoire.** Indicated are Azaguié town and its surrounding villages. Among the 11 schools included in the pre-screening, five were located in the surrounding villages and six were located in the Azaguié town area. The three settings selected for the in-depth studies (i.e. Azaguié M’Bromé/Azaguié Makouguié, rural; Abbé-Bégnini, peri-urban; Azaguié Gare, urban) are emphasised with red asterisks.

The study was conducted within the frame of a multi-country project funded by the Schistosomiasis Consortium for Operational Research and Evaluation (SCORE) to assess the accuracy of a rapid diagnostic urine circulating cathodic antigen (CCA) cassette test for *S. mansoni* infections in different endemicity settings [9]. For the current investigation in Côte d’Ivoire, three *S. mansoni* endemicity settings were to be identified, as follows: (i) low *S. mansoni* endemicity setting (i.e. prevalence in school-aged children of 10–24%); (ii) moderate *S. mansoni* endemicity setting (i.e. prevalence in school-aged children of 25–49%); and (iii) setting where *S. mansoni* and *S. haematobium* are co-endemic. Recent research conducted in the district of Azaguié revealed a variety of schistosome endemicity scenarios [10-12], and hence this district, located approximately 40 km north of Abidjan (Figure 1), was chosen for the current investigation. In September 2010, we carried out a rapid screening in 11 schools. Twenty-five children, aged 8–12 years, were randomly selected by drawing lots in each school. Children provided one stool and one urine sample that were subjected to quality-controlled techniques.

Based on the screening results, three schools that fitted in the prevalence scheme indicated above were selected. All children aged 8–12 years were considered eligible for participation. According to sample size calculations for the overarching SCORE project, around 200 children from existing school lists were randomly selected in each of the three settings and invited to participate in the main study.

### Field procedures

For the main study, in the early morning, right after school lessons had started, children were given plastic containers labelled with unique identification numbers (IDs) and invited to return the containers filled with a fresh morning stool sample (10–50 g) the following day. Upon sample collection, new empty containers were handed out for stool collection the next morning. This procedure was repeated over three days. Catch-up collections were done for two more days, and by then most of the children had submitted three stool samples. Additionally, after the children had provided stool samples, they were given another container and asked to provide a urine sample to be produced between 10:00 and 12:00 hours.

### Laboratory procedures

Stool and urine collections were completed around noon. The samples were transferred to a parasitological laboratory at the Université de Cocody in Abidjan and processed the same day as follows. First, triplicate 41.7 mg Kato-Katz thick smears were prepared from each stool sample [13]. The slides were allowed to clear for at least 30 min before examination under a microscope by one of five experienced laboratory technicians. The number of helminth eggs were counted and recorded for each species separately. Second, from each participant’s second day stool sample, ~1 g of stool was placed into a Falcon tube containing 10 ml of sodium acetate-acetic acid-formalin (SAF) solution, broken and homogenized with a wooden spatula and vigorously shaken. Third, urine samples were shaken and 10 ml filtered using a plastic syringe and filter-holders with 13-mm diameter filter (mesh size 20 μm) (Sefar AG; Heiden, Switzerland). Filters were removed with forceps, placed on microscope slides, a drop of Lugol’s solution added and examined under a microscope. The number of *S. haematobium* eggs was counted and recorded.

Within 6–10 weeks of stool collection, the SAF-fixed samples were subjected to an ether-concentration method, using a standard protocol [14]. In brief, the SAF-fixed stool samples were re-suspended and filtered through a medical gauze placed in a plastic funnel into a centrifuge tube. The tube was centrifuged for 1 min at 500 *g*. After centrifugation, the supernatant was discarded and 7 ml of 0.85% NaCl plus 2–3 ml of ether were added to the remaining pellet. After shaking for 10–30 s, the tube and its contents were centrifuged for 5 min at 500 *g*. Finally, from the four layers formed, the three top layers were discarded. The bottom layer, including the sediment, was examined under a microscope. The number of helminth eggs were counted and recorded for each species separately. Intestinal protozoa were recorded semi-quantitatively as follow: (i) negative (no cysts or trophozoites in the entire sediment); (ii) light (one to five cysts or trophozoites per slide); (iii) moderate (one cyst or trophozoite per observation field at a magnification of x 400 or x 500); and (iv) heavy (more than one cyst or trophozoite per observation field at a magnification of x 400 or x 500) [14].

### Statistical analysis

Data were double entered and cross-checked using EpiInfo version 3.2 (Centers for Disease Control and Prevention; Atlanta, GA, USA). Statistical analyses were carried out using STATA version 10 (Stata Corporation; College Station, TX, USA).

Parasite species-specific data analysis was restricted to those children who had complete data records (i.e. three stool samples examined with triplicate Kato-Katz thick smears for *S. mansoni* and soil-transmitted helminths, one stool sample subjected to an ether-concentration method for helminths and intestinal protozoa, and three urine samples examined with a single urine filtration for *S. haematobium*).

For each individual, arithmetic mean egg counts of the helminths were calculated from the nine Kato-Katz thick smears and three urine filtration readings. Helminth infection intensities were expressed as eggs per gram of stool (EPG) and eggs/10 ml of urine (for *S. haematobium*) and classified into light, moderate and heavy infection intensities, according to thresholds put forth by the World Health Organization (WHO) [1,15]. Helminth infection intensities were also estimated at the setting level, using the group’s arithmetic mean faecal egg counts [16].

Dichotomous data are presented as proportion in the pre-screening as well as for the three main study settings. For the latter settings, multivariable logistic regression models were fitted for each parasite. The prevalence of a respective parasite was used as dependent variable (binary variable: present/absent) and the prevalence of each other parasite, sex (binary variable: male/female), age (continuous variable) and the study settings (categorical variable) were used as explanatory variables. As preparatory analysis for assessment of our multivariable logistic regressions, in each setting, univariable logistic regression was used to assess the association between each dependent variable (parasite infection) and each covariate (i.e. sex, age, village and other parasites). Subsequently, interactions between sex, age and village were assessed for each dependent variable. Next, village effects were assessed by considering a clustered structure in the models (xtlogit command in STATA). That was, we considered a random intercept for each village in the logistic regression model. Village effects for the respective parasites were fitted in each final model. At the end, a backward stepwise elimination procedure was applied, including interactions and village effect to determine significant association between parasitic infections and between parasitic infections and study setting. The explanatory variable with the highest p-value in the multivariable logistic regression model was eliminated before re-running the multivariable logistic regression model and these iterations of elimination were continued as long as the values of the Akaike information criterion (AIC) of the new models were decreasing. A similar approach has already been successfully used in other studies [17]. A p-value below 0.05 was considered as statistically significant.

## Results

### Pre-screening

Table 1 shows the results of the initial screening carried out in 11 schools in the Azaguié district, south Côte d’Ivoire. According to triplicate Kato-Katz thick smears derived from a single stool sample obtained from 25 children per school, the prevalence of *S. mansoni* ranged between nil and 88%. Abbé-Bégnini with a *S. mansoni* prevalence of 16% was the only low endemicity school, whereas the three schools in Azaguié Gare were determined as moderately endemic (*S. mansoni* prevalences ranging between 25% and 49%). *S. haematobium* infections, determined by single urine filtrations, were found in seven of the 11 schools. In five of these schools, only one to three children were infected. However, in Azaguié M’Bromé and Azaguié Makouguié, more than half of the children were infected with *S. haematobium*. Hence, the settings Abbé-Bégnini, Azaguié Gare and Azaguié M’Bromé/Azaguié Makouguié were selected, as they fitted SCORE’s predefined classifications of low, moderate and mixed endemicity, respectively.

**Table 1:** **Prevalence of*S. mansoni*and*S. haematobium*, as assessed in an initial screening carried out in 11 schools in Azaguié district, south Côte d’Ivoire in September 2010**

**School**	**No. (%) of infected children**		**Endemicity**^**a**^
	***S. mansoni***	***S. haematobium***	
Abbé-Bégnini	4 (16)	0	Low
Achiékoua	0	0	Not selected
Ahoua 1	14 (56)	1 (4)	Not selected
Ahoua 2	14 (56)	1 (4)	Not selected
Ahoua 3	15 (60)	2 (8)	Not selected
Azaguié Gare 1A	9 (36)	0	Moderate
Azaguié Gare 2A	7 (28)	0	Moderate
Azaguié Gare 2B	9 (36)	3 (12)	Moderate
Bambou	11 (44)	1 (4)	Not selected
Azaguié M’Bromé	17 (68)	14 (56)	Mixed
Azaguié Makouguié	22 (88)	13 (52)	Mixed

^a^ Endemicity was set according to SCORE guidelines: prevalence of *S. mansoni* between 10% and 24% indicates low endemicity, prevalence of *S. mansoni* between 25% and 49% was considered moderate endemicity, co-existence of *S. mansoni* and *S*. *haematobium* indicates mixed endemicity.

In each school, the prevalence was assessed among 25 randomly selected children, aged 8–12 years. One stool sample was examined with triplicate Kato-Katz thick smears to determine the prevalence of *S. mansoni*, whereas one urine sample was subjected to a single filtration to assess the prevalence of *S. haematobium*.

### Characteristics of study settings and population

Adhering to SCORE guidelines, Abbé-Begnini (low endemicity *S. mansoni* setting), Azaguié Gare (moderate endemicity *S. mansoni* setting) and Azaguié M’Bromé/Azaguié Makouguié (mixed *S. mansoni*-*S. haematobium* setting) were selected for an in-depth appraisal of school-aged children’s helminth and intestinal protozoan infection profiles. Interestingly, Azaguié M’Bromé/Azaguié Makouguié, Abbé-Bégnini and Azaguié Gare (Figure 1) were located in rural, peri-urban and urban areas, respectively. The proportions of children with complete data records from the sampling of stool and urine were 77.3% in Azaguié M’Bromé/Azaguié Makouguié, 62.4% in Abbé-Bégnini and 59.1% in Azaguié Gare.

Azaguié M’Bromé and Azaguié Makouguié, two small villages located approximately 9 and 12 km west of Azaguié town, are typical rural settings. These two villages are difficult to access via gravel roads that are infrequently used by private transport. People are mainly engaged in subsistence farming. There is no tap water supply and households lack sanitation facilities, and hence the population practices open defecation. In each village, there is a primary school. Additionally, in Azaguié M’Bromé, there is a primary health care centre.

Abbé-Bégnini is a peri-urban village located approximately 5 km east of the centre part of Azaguié town. The village is reachable on a tarmac road by private and public transport. Subsistence farming is the main source of income. Sanitation coverage is low and inhabitants depend on open surface water from the nearby river for household use and on two water pumps for drinking and cooking. There is one primary health care centre.

Azaguié Gare is an urban neighbourhood of Azaguié town, which has excellent transport connections to Abidjan (bus, car and train). The population mainly consists of civil servants, artisans, traders and some farmers. Most of the inhabitants have access to tap water and only few use public man-made wells. Latrines with cemented slabs or flush toilets are common. The town has several private primary health care centres and a public secondary health facility.

### Intestinal parasitic infections

**Figure 2:**
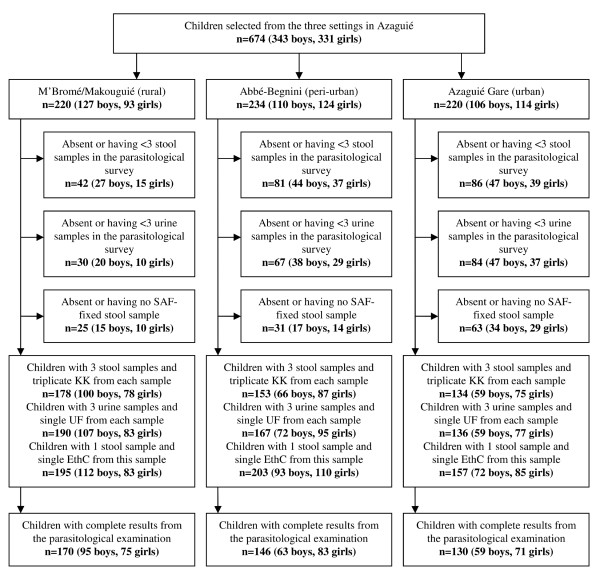
**Flow chart detailing the study participation and adherence.** Children were selected from three different settings in Azaguié district, south Côte d’Ivoire in October and November 2010. KK, Kato-Katz technique; UF, urine filtration method; EthC, ether-concentration method.

Figure 2 shows the study adherence. Overall, 170, 146 and 130 schoolchildren had complete parasitological data in Azaguié M’Bromé/Azaguié Makouguié, Abbé-Bégnini and Azaguié Gare, respectively. Table 2 shows the infection prevalence of intestinal parasites in the main study, stratified by setting. In rural Azaguié M’Bromé/Azaguié Makouguié, very high prevalences of both *S. mansoni* and *S. haematobium* were found (91.8% and 65.3%, respectively). *T. trichiura* was the predominant soil-transmitted helminth (56.5%). While hookworm was also common (44.7%), the prevalence of *A. lumbricoides* was considerably lower (11.2%). *E. coli* with a prevalence of 31.8% was found to be the predominant intestinal protozoon infection, followed by *Endolimax nana* (28.8%), *Blastocystis hominis* (15.9%) and *G. intestinalis* (8.8%).

**Table 2:** Prevalence of helminths and intestinal protozoa infections in three settings of Azaguié district, south Côte d’Ivoire in October and November 2010

**Intestinal parasite**	**Study setting**^**a**^					
	**Azaguié M’Bromé/Azaguié Makouguié (rural)**		**Abbé-Begnini (peri-urban)**		**Azaguié Gare (urban)**	
	**No. of infected**	**% (95% CI)**	**No. of infected**	**% (95% CI)**	**No. of infected**	**% (95% CI)**
**Helminths**						
*Schistosoma mansoni*	156	91.8 (87.6–95.9)	48	32.9 (25.2–40.6)	69	53.1 (44.4–61.8)
*Schistosoma haematobium*	111	65.3 (58.1–72.5)	6	4.1 (0.1–7.4)	1	0.8 (0–2.0)
*Trichuris trichiura*	96	56.5 (48.9–63.9)	10	6.9 (2.7–10.9)	20	15.4 (9.1–21.7)
Hookworm	76	44.7 (37.2–52.3)	60	41.1 (33.0–49.2)	27	20.8 (13.7-27.8)
*Ascaris lumbricoides*	19	11.2 (6.4–15.9)	10	6.9 (2.7–10.9)	17	13.1 (7.2–18.9)
**Intestinal protozoa**						
*Entamoeba coli*	54	31.8 (24.7–38.8)	39	26.7 (19.5–33.9)	33	25.4 (17.8–32.9)
*Endolimax nana*	49	28.8 (21.9–35.7)	36	24.7 (17.6–31.7)	55	42.3 (33.7–50.9)
*Blastocystis hominis*	27	15.9 (10.7–22.3)	14	9.6 (4.8–14.4)	14	10.8 (5.4–16.2)
*Giardia intestinalis*	15	8.8 (4.5–13.1)	11	7.5 (3.2–11.9)	12	9.2 (4.2–12.3)
*Chilomastix mesnili*	14	8.2 (4.1–12.4)	7	4.8 (1.3–8.3)	6	4.6 (0.9–8.3)
*Entamoeba histolytica/E. dispar*	14	8.2 (4.1–12.4)	12	8.2 (3.7–12.7)	7	5.4 (1.5–9.3)
*Iodamoeba bütschlii*	9	5.3 (1.9–8.7)	8	5.5 (1.7–9.2)	10	7.7 (3.1–12.3)
*Entamoeba hartmanni*	5	2.9 (0.4–5.5)	8	5.5 (1.7–9.2)	9	6.9 (2.5–11.3)

^a^ n = 170 (rural setting), n = 146 (peri-urban setting), n = 130 (urban setting).

Diagnosis of *S. mansoni* and soil-transmitted helminths were based on nine Kato-Katz thick smears (three stool samples, each subjected to triplicate Kato-Katz). Diagnosis of *S. haematobium* was based on three urine samples, each subjected to a single filtration. Diagnosis of intestinal protozoa was based on a single stool sample fixed in SAF that was examined with an ether-concentration technique.

CI, confidence interval.

In peri-urban Abbé-Bégnini, hookworm and *S. mansoni* showed the highest prevalences, 41.1% and 32.9%, respectively. The prevalence of *S. haematobium*, *T. trichiura* and *A. lumbricoides* were below 10%. *E. coli* and *E. nana* were the predominant intestinal protozoa with respective prevalences of 26.7% and 24.7%.

In urban Azaguié Gare, more than half (53.1%) of the children harboured *S. mansoni*. The prevalences of hookworm, *T. trichiura* and *A. lumbricoides* were 20.8%, 15.4% and 13.1%, respectively. Only one child (0.8%) was found with *S. haematobium* eggs in the urine. In this setting, *E. nana* was the predominant intestinal protozoon species (42.3%), followed by *E. coli* (25.4%) and *B. hominis* (10.2%).

### Infection intensities

Infection intensities, expressed as group arithmetic mean faecal egg counts, showed some heterogeneity between settings (Table 3). Infection intensity classes for helminths and intestinal protozoa, stratified by setting, are summarised in Table 4. In rural Azaguié M’Bromé/Azaguié Makouguié, more than two-thirds of *S. mansoni*-infected children had either a moderate (35.2) or a heavy (41.7%) infection. *S. haematobium*, hookworm, *T. trichiura* and *A. lumbricoides* infections were mainly of light intensities. Most of the children were heavily infected with intestinal protozoa. In peri-urban Abbé-Bégnini, 97.0% of the infected schoolchildren showed light helminth infection intensities. Intestinal protozoan infection intensities showed no clear intensity patterns. In urban Azaguié Gare, all helminth infections were of light intensities. Intestinal protozoan infections were light (58.4%) or moderate (36.5%) and only 13 children showed a heavy infection.

**Table 3:** Arithmetic mean helminth eggs per gram of stool (EPG), stratified by setting

	**Azaguié M’Bromé/Azaguié Makouguié (rural)**		**Abbé-Bégnini (peri-urban)**		**Azaguié Gare (urban)**	
	**Arithmetic mean**	**95% CI**	**Arithmetic mean**	**95% CI**	**Arithmetic mean**	**95% CI**
*Schistosoma mansoni*	482.7	388.1–577.4	17.4	0.0–38.9	62.4	36.2–88.5
*Schistosoma haematobium*^a^	19.4	13.3–25.5	2.3	0.4–4.2	1.5	0.5–2.6
*Ascaris lumbricoides*	10.3	0.2–20.4	46.3	5.5–87.2	175.4	0.0–474.1
*Trichuris trichiura*	136.4	81.7–191.1	9.4	0.0–23.2	5.9	3.1–8.8
Hookworm	49.1	17.9–80.2	56.7	23.8–89.6	6.6	3.3–9.8

^a^ infection intensity expressed as eggs per 10 ml of urine.

CI, confidence interval.

**Table 4:** **Categories of helminths and intestinal protozoan infection intensities, stratified by setting.** Helminth infection intensities were categorized according to the classification of WHO [1,15] and intestinal protozoan infection intensities were classified as described elsewhere [14]

**Parasite**	**Study setting**								
	**Azaguié M’Bromé/Azaguié Makouguié (rural)**			**Abbé-Begnini (peri-urban)**			**Azaguié Gare (urban)**		
	**Light (%)**	**Moderate (%)**	**Heavy (%)**	**Light (%)**	**Moderate (%)**	**Heavy (%)**	**Light (%)**	**Moderate (%)**	**Heavy (%)**
**Helminths**									
*Schistosoma mansoni*	36 (23.1)	55 (35.2)	65 (41.7)	46 (95.8)	1 (2.1)	1 (2.1)	47 (68.1)	17 (24.6)	5 (7.2)
*Schistosoma haematobium*^a^	91 (82.0)	--	20 (18.0)	5 (83.3)	--	1 (16.7)	0	--	1 (100)
Hookworm	75 (100)	0	0	59 (98.3)	1 (1.7)	0	27 (100)	0	0
*Trichuris trichiura*	95 (95.0)	5 (5.0)	0	10 (90.9)	1 (9.1)	0	20 (100)	0	0
*Ascaris lumbricoides*	19 (100)	0	0	10 (100)	0	0	16 (94.1)	1 (5.9)	0
**Intestinal protozoa**									
*Entamoeba coli*	11 (20.4)	18 (33.3)	25 (46.3)	7 (17.9)	14 (35.9)	18 (46.2)	15 (50.0)	12 (40.0)	3 (10.0)
*Endolimax nana*	14 (28.6)	9 (18.4)	26 (53.1)	7 (19.4)	13 (36.1)	17 (47.2)	26 (46.4)	23 (41.1)	7 (12.5)
*Blastocystis hominis*	8 (33.3)	5 (20.9)	11 (45.8)	7 (50.0)	3 (21.4)	1 (7.1)	9 (64.3)	5 (35.7)	0
*Giardia intestinalis*	1 (6.7)	4 (26.7)	10 (66.7)	1 (9.1)	3 (27.3)	7 (63.6)	8 (66.7)	3 (25.0)	1 (8.3)
*Chilomastix mesnili*	2 (14.3)	4 (28.6)	8 (57.1)	1 (14.3)	1 (14.3)	5 (71.4)	3 (50.0)	2 (33.3)	1 (16.7)
*Entamoeba histolytica/E. dispar*	4 (28.6)	8 (57.1)	2 (14.3)	3 (25.0)	4 (33.3)	5 (41.7)	6 (66.7)	2 (22.2)	1 (11.1)
*Iodamoeba bütschlii*	0	3 (33.3)	6 (66.7)	4 (50.0)	1 (12.5)	3 (33.3)	8 (80.0)	2 (20.0)	0
*Entamoeba hartmanni*	1 (20.0)	1 (20.0)	3 (60.0)	4 (50.0)	2 (25.0)	2 (25.0)	8 (88.9)	1 (11.1)	0

^a^ Infection intensity cut-off for *S. haematobium* are 1–49 eggs per 10 ml of urine (light) and ≥50 eggs per 10 ml of urine (heavy).

The study was carried out in Azaguié, south Côte d’Ivoire in October and November 2010. *S. mansoni* and soil-transmitted helminth infection intensities were based on the reading of nine Kato-Katz thick smears. Intestinal protozoan infection intensities were based on one SAF-fixed stool sample subjected to an ether-concentration technique. Three urine filtrations were done to determine infection intensities of *S. haematobium*.

### Multiparasitism

**Figure 3:**
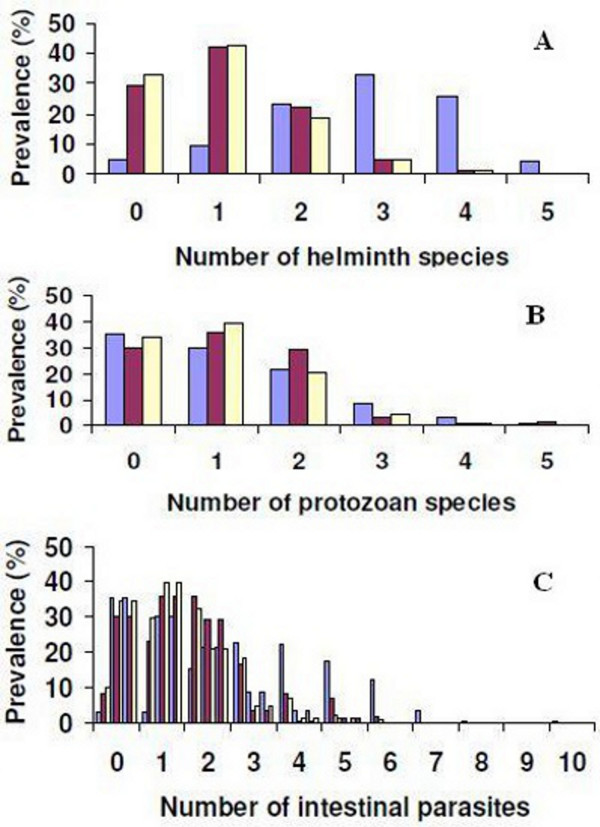
**Multiple intestinal parasitic infections among schoolchildren aged 8–12 years in three settings of Azaguié district, south Côte d’Ivoire, in October and November 2010.** Blue bars indicate rural Azaguié M’Bromé/Azaguié Makouguié; purple bars indicate peri-urban Abbé-Bégnini; and yellow bars indicate urban Azaguié Gare. (**A**) Number of helminth species diagnosed per child; (**B**) number of intestinal protozoa species diagnosed per child; (**C**) number of intestinal parasites (helminths and intestinal protozoa) diagnosed per child.

Figure 3 shows the patterns of multiple species intestinal parasite infections, stratified by setting. In the rural setting, the prevalence of schoolchildren with single, dual or multiple species helminth infection was 9.2%, 22.4% and 61.6%, respectively. In the peri-urban setting, 42.1%, 22.1% and 6.2% of the children harboured one, two or at least three helminth species concurrently. In the urban setting, single helminth species infections were most common (41.2%), whereas dual species helminth infections were found in 18.2% of the children investigated (Figure 3A).

Multiple species intestinal protozoan infections were assessed with a single ether-concentration test. In rural Azaguié M’Bromé/Azaguié Makouguié, 29.9% of the children harboured single species intestinal protozoan infections, whereas 21.3% harboured two species of intestinal protozoa concurrently. An infection with three or more intestinal protozoan species was found in 12.5% of the children. In peri-urban Abbé-Bégnini, 36.5%, 29.3% and 5.1% showed single, dual or multiple species intestinal protozoan infections. In urban Azaguié Gare, 40.1% harboured single species intestinal protozoan infections, whereas dual or multiple species infections were found in 21.6% and 5.4%, respectively (Figure 3B).

Considering all intestinal parasite species (i.e. helminths and intestinal protozoa), the prevalence of single, dual and multiple species infections in the rural setting was 2.9%, 15.2% and 79.5%, respectively. Two schoolchildren harboured eight or 10 intestinal parasites concurrently. In the peri-urban setting, 39.4%, 32.4% and 28.2% of the schoolchildren harboured single, dual and multiple species infections, respectively. In the urban setting, the prevalence of single, dual and multiple intestinal parasite infections was 23.0%, 35.7% and 33.3%, respectively (Figure 3C).

### Parasite associations

All significant associations (p <0.05) between intestinal parasites, sex, age and setting resulting from the multivariable logistic regression models are summarised in Table 5. *S. mansoni* infection showed significant positive associations with *S. haematobium* (odds ratio (OR) = 4.81, 95% confidence interval (CI) = 1.79–12.93) and *T. trichiura* (OR = 2.74, 95% CI = 1.34–5.60). The odds of being infected with *S. mansoni* increased with age (OR = 1.55, 95% CI = 1.29–1.87). *A. lumbricoides* was significantly associated with *T. trichiura* (OR = 4.24, 95% CI = 1.96–9.19) and hookworm (OR = 2.34, 95% CI = 1.14–4.41). Boys were more likely to be infected with hookworm than girls (OR = 1.87, 95% CI = 1.24–2.81). *E. coli* was significantly associated with *S. haematobium* (OR = 1.73, 95% CI = 1.09–2.72).

**Table 5:** Significant associations of intestinal parasitic infections among schoolchildren in Azaguié district, south Côte d’Ivoire, in October and November 2010 (n = 446)

**Parasite**	**Association**	**Adjusted OR (95% CI)**
**Schistosomiasis**		
*Schistosoma mansoni*	*S. haematobium*	4.81 (1.79–12.93)
	*T. trichiura*	2.74 (1.34–5.60)
	Age	1.55 (1.29–1.87)
*Schistosoma haematobium*	*S. mansoni*	4.09 (1.65–10.84)
**Soil-transmitted helminths**		
*Ascaris lumbricoides*	*T. trichiura*	4.24 (1.96–9.19)
	Hookworm	2.34 (1.14–4.41)
	Rural setting	0.31 (0.13–0.73)
*Trichuris trichiura*	*S. mansoni*	2.89 (1.42–5.91)
	*A. lumbricoides*	4.14 (1.90–8.99)
Hookworm	*A. lumbricoides*	3.03 (1.56–5.87)
	Sex	1.87 (1.24–2.81)
**Intestinal protozoa**		
*Endolimax nana*	*B. hominis*	2.39 (1.33–4.29)
	Peri-urban setting	0.44 (0.26–0.74)
	Rural setting	0.50 (0.31–0.82)
*Entamoeba coli*	*S. haematobium*	1.73 (1.09–2.72)
*Blastocystis hominis*	*E. nana*	2.44 (1.37–4.35)

Associations between a particular parasite (as binary variable; reference, absence) as dependent variable and age (as continuous variable), sex (as binary variable; reference, female), study setting (as categorical variable; reference, urban setting) and any of the remaining parasites (as binary variable; reference, absence) were analysed with multivariable logistic regression models, performing a stepwise backward elimination procedure.

CI, confidence interval; OR, odds ratio.

## Discussion

We studied patterns of intestinal parasite infections (i.e. schistosomes, soil-transmitted helminths and intestinal protozoa) in school-aged children in three settings of south Côte d’Ivoire; a rural, peri-urban and urban area. An important aspect of parasitic disease control programmes is the ability to readily identify and reach people at highest risk of infection and associated morbidity. Often, the poorest people are the least accessible ones living in remote rural areas, and hence they are at highest risk of parasitic infection and other conditions of ill-health [18-20].

In this study, the assessment of children’s prevalence and intensity of *S. mansoni* and soil-transmitted helminth infections was based on nine Kato-Katz thick smears derived from three stool samples. The day-to-day and intra-specimen variation in helminth egg output that compromise the sensitivity of the Kato-Katz technique, especially in areas of low infection intensity, is overcome by such a rigorous diagnostic approach [21-23]. However, multiple stool sampling to increase the sensitivity of the Kato-Katz technique for helminth diagnosis might result in reduced compliance. Indeed, particularly in the urban setting, compliance for providing all three stool samples was considerably lower than in the rural and peri-urban settings and this might have introduced some bias.

Another aspect of our study worth highlighting is the following. After our pre-screening based on a single stool sample, and then employing a more rigorous diagnostic approach, the previously anticipated *S. mansoni* endemicity levels were considerably overrun. As predicted by mathematical modelling [24] and confirmed in field studies, repeated stool sampling with multiple Kato-Katz thick smears prepared from individual stool samples, result in considerable increases of the observed prevalence of *S. mansoni*[21,23,25]. The same observations have also been made for soil-transmitted helminths [22,26]. It follows that if rapid screenings are performed to reliably select a study setting or treatment scheme according to prevalence estimates, more sensitive diagnostic tools as alternatives to a rigorous sampling approach (i.e. collecting multiple stool samples and examining them with multiple Kato-Katz thick smears or other methods) are needed, particularly in areas with low transmission rates.

The current study confirms that schoolchildren from a rural setting are at higher risk of helminth infections than their counterparts living in peri-urban or urban settings. The remoteness of the rural setting, characterized by the absence of key infrastructures (e.g. tarmac road, health facilities, tap water and basic sanitation) play important roles. Our observations are in line with previous epidemiological surveys. Indeed, unsafe hygiene, water and sanitation and inadequate management of the environment exacerbate parasite infections in general, and helminth infections in particular [7,19,20,27,28]. Greatest differences between the prevalence of helminth infections, as a function of the study setting, were found with regard to the two schistosome species. Besides socioeconomic risk factors, the transmission of *S. mansoni* and *S. haematobium* is governed by intermediate host snails [6,8,29], and hence the availability of suitable snail habitats seem to vary considerably between the three settings even at this small-scale. While it is commonly believed that schistosomiasis is a “rural disease”, some studies have shown high prevalence in urban settings [30]. Hence, detailed malacological investigations are needed to deepen our understanding of the epidemiology of schistosomiasis with regard to the level of urbanization. Interestingly, similar hookworm prevalences were found in the rural and peri-urban settings. This observation could be explained by the fact that the behaviour of the schoolchildren with regard to hygiene and faecal management in particular is similar. Since open defecation is widely practiced in these communities, efforts must be made to improve sanitation, which in turn will have major ramification on other neglected tropical diseases such as amoebiasis and giardiasis [28,31].

Interestingly, the prevalence of intestinal protozoan infections among schoolchildren was found to be similar in the three settings. This might indicate that hygiene related to food consumption among schoolchildren is similar and needs to be improved. The two predominant intestinal protozoan species in the three settings under investigation are *E. coli* and *E. nana*. This observation is in agreement with previous studies done in different parts of Côte d’Ivoire [10,31,32].

Regarding intestinal parasite infection intensities, with the exception of *S. mansoni* in the rural setting, all other helminth infections were of light intensities whatever the setting. The observed moderate and heavy *S. mansoni* infections in the rural setting might be explained by high transmission in the absence of preventive and curative measures. Indeed, we are not aware of large-scale prior deworming activities in the district of Azaguié. There was a tendency of infection intensities of intestinal protozoa to decrease from rural to urban settings. Socioeconomic factors and educational attainment increase from rural to urban settings, and hence might explain the observed decrease in the intensities of intestinal protozoa.

Our study also confirms that multiparasitism is pervasive as observed elsewhere in sub-Saharan Africa and Asia [17,33-37]. We found two children in the rural setting harbouring eight or 10 different intestinal parasite species concurrently. This high number of parasite species in a single host is an alarming situation, as multiple species infection may increase susceptibility to other parasites [38-40]. Associations between different parasite species, as well as the influence of age, sex and study setting, have been assessed in the current investigation. Of particular note is the strong positive association between *S. mansoni* and *S. haematobium* with an adjusted OR above four. Moreover, we found a significant association between *S. haematobium* and *E. coli*, which has not been described in the literature before. Boys and girls were at the same level of exposure to helminths and intestinal protozoa, except for hookworm, as boys showed significantly higher infection prevalence than girls. Interestingly, a previous study carried out by Keiser *et al.* (2002) in western Côte d’Ivoire found that hookworm infections were significantly more often diagnosed in girls [17,35]. Behavioural and socioeconomic factors might explain this observed difference. Our study is in line with previous investigations that the risk of becoming infected with *S. mansoni* increases with age in children [41,42]. To date, the effects of parasite interactions on the human body remain poorly understood. Without a deeper understanding of such parasite interactions, the effectiveness of parasitic disease control programmes are compromised.

## Conclusion

Continued and concerted efforts should be made by control programmes to reach rural school-aged children and other high-risk groups in the most remote areas, as they are the least accessed ones whose health and wellbeing is insufficiently accounted for by policy makers. Improvement of safe water supply and sanitation facilities by the construction of toilets could significantly reduce the burden of parasitic diseases in the rural and peri-urban settings studied here. In addition, particular importance might be given to health education at the district level. Control programmes should carefully consider the benefits of truly integrated (i.e. inter-programmatic and inter-sectoral) strategies. The findings of the present study may provide useful information for such integrated strategies to overcome the public health burden of intestinal parasitic infections in the Azaguié district in south Côte d’Ivoire in particular, and in other settings of the humid tropics.

## Competing interests

The authors declare that they have no conflict of interest concerning the work reported in this paper.

## Authors’ contributions

JTC, TF, SK, JU and EKN designed the study; JTC, KDS, SK, MO and EKN implemented the study; JTC managed the data; JTC, TF, SK, DH, JU and EKN analysed and interpreted the data; JTC wrote the first draft of the paper; TF, SK, JU and EKN revised the paper. All authors read and approved the final version of the manuscript before submission.
